# DTI-ALPS as a marker of glymphatic function: Review of the literature findings, methodology, and interpretation

**DOI:** 10.1016/j.ynirp.2026.100394

**Published:** 2026-08-06

**Authors:** Jerome J. Maller, Vincent A. Magnotta

**Affiliations:** Magnetic Resonance Research Facility, Department of Radiology, University of Iowa, Iowa, United States

## Abstract

The glymphatic system (GS) describes how the brain clears toxins and metabolic waste. Accumulation of these elements can contribute to onset and severity of brain disorders, so the importance of measuring the GS is vital to understanding how neurological disease may occur and potentially lead to developing preventative treatments. A simple technique referred to as Diffusion Tensor Image Analysis ALong the Perivascular Space (DTI-ALPS) was developed as a non-invasive MRI marker of GS functioning. However, various aspects of its measurement and putative interpretation have led to critiques of the technique and discussion focused upon whether it is truly a measure of GS. We located 393 DTI-ALPS articles with an exponential growth during the past few years. The most studied conditions where this technique has been employed was neurodegenerative diseases, followed by many other conditions such as sleep disorders, epilepsy, stroke, and mood disturbances. While most studies found a significant association with DTI-ALPS and their cohorts, there has been significant discussion related to the interpretation of the DTI-ALPS index and the mechanisms underlying changes in this value. The majority of studies found significant associations between DTI-ALPS and disease severity, although issues related to differences in methodologies are important to consider. Finally, the methods employed to generate the DTI-ALPS measurements as well as the variation in these approaches are summarized. DTI-ALPS is mathematically governed by radial diffusivity asymmetry and axonal geometry, and it serves as a highly sensitive but non-specific marker of white matter microstructural damage and neuroinflammation. Retrospectively applicable and highly reproducible, DTI-ALPS offers substantial translational potential for low-cost, large-scale clinical screening and longitudinal trial stratification when paired with other biomarkers.

## 1 Introduction

**Fig. 1:**
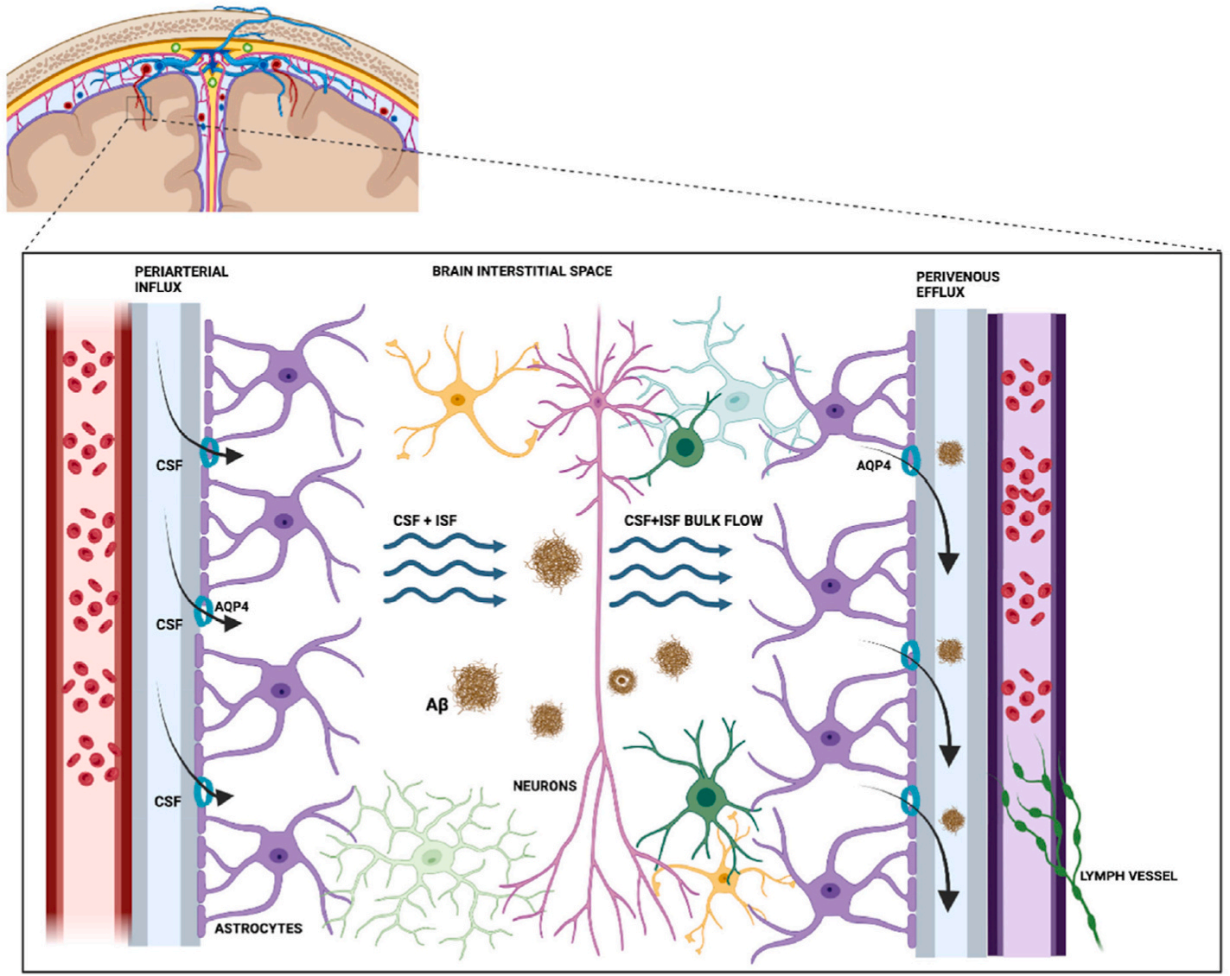
Glymphatic system role in cerebrospinal fluid (CSF) circulation and waste clearance in the brain. CSF enters the brain along periarterial spaces, facilitated by aquaporin-4 (AQP4) water channels located on astrocyte end feet. The CSF then mixes with interstitial fluid (ISF) in the brain interstitial space, aiding in the removal of soluble amyloid-beta (Ab) and other metabolic waste products. This mixture is driven through the brain tissue by bulk flow and exits via perivenous spaces, ultimately being transported to lymphatic vessels for elimination. (From (Botta et al., 2025)).

Over 10 years ago, Iliff and colleagues conducted a simple yet elegant study which proposed that the glymphatic (glial-lymphatic) system (GS; Fig. 1) reflects the exchange of fluid between the subarachnoid CSF and the interstitial space of the brain parenchyma via the perivascular space and intervening aquaporin 4 (AQP4) channels (Iliff et al., 2012). Their work began efforts to use non-invasive imaging techniques to study how the brain clears toxins and metabolic waste (Khalafi et al., 2025).

AQP4 is a water channel positioned in the astrocyte membrane and is thought to be the primary driver of glymphatic clearance. That is, it is described as a brain waste clearance system (Andica et al., 2023) that also facilitates nourishment and local transmission in the brain by distributing glucose to neurons and astrocytes as well as delivering lipids and apolipoprotein E (Rasmussen et al., 2022). Specifically, CSF moves from the ventricles and subarachnoid spaces (through the periventricular spaces, PVS) into the parenchyma which then promotes interstitial fluid (ISF) and metabolic waste fluxes toward the perivenous spaces surrounding the deep veins (Mestre et al., 2020). Specifically, CSF and interstitial fluid (ISF) are cleared along paravenous drainage pathways via AQP4 water channels in the astrocyte's feet (Ulloa et al., 2025). It has been demonstrated that GS activity increases during sleep and is accompanied by expansion of the interstitial space (Xie et al., 2013) which facilitates the exchange of CSF and ISF, hence enhancing the clearance of neurotoxic proteins compared to the awake state (Yun et al., 2025). Agarwal et al. described this finding as a “groundbreaking shift in neuroscience” (Agarwal, 2025). Changes to the AQP4 glymphatic clearance has been associated with a buildup of various proteins including amyloid-β (Aβ) and α-synuclein, which are associated with Alzheimer's Disease (AD) (Barichello de Quevedo et al., 2023) and Parkinson's Disease (PD) (Buccellato et al., 2022) respectively.

Immunofluorescence and dynamic contrast-enhanced (DCE) MRI are most direct imaging methods used to study the GS by revealing the degree of perivascular AQP4 polarization and extent of CSF-ISF exchange activity (Kress et al., 2014; Iliff et al., 2013)}, but these are invasive techniques as they require injection of contrast. A recently introduced technique called Diffusion Tensor Image Analysis ALong the Perivascular Space (DTI-ALPS) index (herein referred to as ALPS index) is purported to be a non-invasive MRI marker of glymphatic functioning (Taoka et al., 2017).

**Fig. 2:**
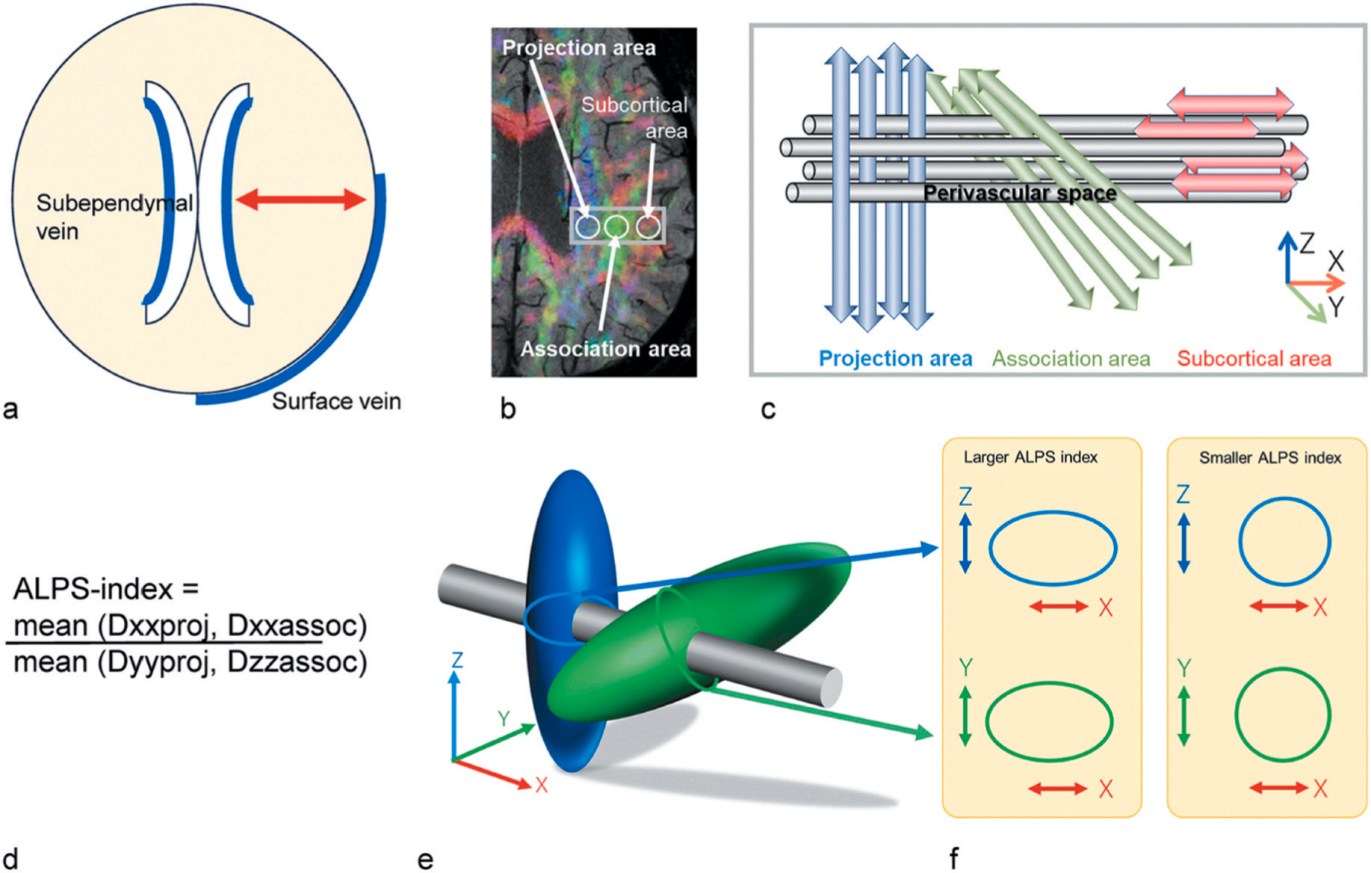
Concept of the DTI-ALPS method. The assumed direction of mass transport within the brain parenchyma is shown in a. For waste material to reach the superficial cerebral veins or the subependymal veins, it must travel in the x-direction, indicated by the red arrow. In actual brain tissue, the medullary vessels also run in this x-direction. The positional relationship between the running direction of major fibers in the white matter outside the lateral ventricles and the location of the medullary vessels and perivascular space is shown in b and c. At this location, projection fibers and medullary vessels and association fibers and medullary vessels are in an orthogonal position to each other. This makes it possible to evaluate the diffusion component in the direction of the medullary vessels by eliminating the influence of the strong diffusion component of the projection and association fibers. The formula for the ALPS index is shown in d. The ratio of the diffusion component perpendicular to both the projection fiber and the medullary vessels and the diffusion component perpendicular to both the association fiber and the medullary vessels is employed as an internal reference to evaluate the diffusion component in the direction of the medullary vessels. The image of the diffusion tensor ellipsoid is shown in e. The major axes of the ellipsoid correspond to projection fibers (blue) and association fibers (green), respectively. The small oval in e is the image of the ALPS-index. The oval that is large in the x-direction corresponds to a large ALPS-index, as shown in f. DTI-ALPS, Diffusion Tensor Image Analysis aLong the Perivascular Space. (From (Taoka et al., 2024)).

The ALPS index is generated by placing regions of interest (ROIs) at the level of the upper corner of lateral ventricle body in the projection and association fiber tracts (Fig. 2). In this region water diffusion is oriented primarily along the fiber tracts and restricted in the other directions. Simply put, the ALPS index reflects the ratio of water diffusivity along the perivenous space to diffusivity along other non-fiber-running directions (1). To make this measurement, Taoka et al. (2017) proposed the following equation

(1) ALPS index = (Dxx_proj_ + Dxx_assoc_)/(Dyy_proj_ + Dzz_assoc_)

An ALPS index close to one is thought to represent minimal glymphatic function and the ALPS index is thought to be positively associated with glymphatic function, that is, the ALPS index increases with increased glymphatic function (Chen et al., 2025a). The range for this value has been shown to be approximately 1.3-2 in younger participants and decrease with age (Lin et al., 2025; Wong et al., 2025; Yin et al., 2024).

While there are ongoing efforts to comprehensively investigate the workings and integrity of the GS, the ALPS index method is posited as a straightforward and relatively accurate indicator of GS efficiency, which requires only single shell DTI data and relatively low b-value (b = 1000). As such, there has been a steady increase in the variety of clinical conditions which have utilized the ALPS index given the large amount of neuroimaging data that has been collected using protocols that obtain such data. However, there are differences between size and specific placement of ROIs reported highlighting the need for standardized ROI selection approaches (Qiu et al., 2025). Given the simplicity of the measurement, additional measures may be needed to generate a more meaningful assessment of the GS. For example, Schilling et al. found that the ALPS index was significantly influenced by white matter (WM) microstructure, including fiber crossings, undulations, and dispersion (Schilling et al., 2025). Hence, the ALPS index may not provide a direct measure of glymphatic function but rather reflect underlying axonal geometry. Furthermore, heterogeneity in medullary vein orientation may challenge the assumption that PVS consistently aligns with the left-right axis in the region where the ALPS index is computed. These aspects underlie the current debate as to whether such a simplistic method accurately provides a measure of GS efficiency and whether it is representative of the GS locally or of the whole brain-wide.

The highly referenced ROI locations utilized by Liu et al. (2024) and Clark et al. (2024) have different ROI sphere coordinates from which the ALPS index is derived. To evaluate the dependence on ROI placement and size, Taoka et al. (2022) used ROIs of various sizes and shapes (spheres, squares, cubes) within the regions described in the original reference (Taoka et al., 2017) to calculate the ALPS index and found that the reproducibility (as measured by ICC; intra-class correlation) ranged from 0.542 to 0.932 based on ROI location and size with larger ROIs providing higher ICC. In addition to ROI position and size, studies have different approaches for mapping the ROIs onto the diffusion weighted images. This has included mapping the ROIs to subject space as well as warping the diffusion weighted images into the template space where the ROIs have been defined. The studies have generally used one the following approaches to measuring the ALPS index after creating the ROIs on a template (usually the JHU-ICBM-FA-1mm) and registering the subject scan to the template. Below are the approaches described with the corresponding FSL software commands (Jenkinson et al., 2012) but other image registration tools have been used as well:

1. FLIRT (linear transformation) of the subject's FA map to template FA map followed by APPLYWARP to register the subject's voxel level tensor estimate to the template to generate and calculate the ALPS index in template space
2. FLIRT (linear transformation) of the subject's FA map to template FA map → FNIRT (non-linear transformation) → APPLYWARP (to apply the non-linear transformation matrix) to register the subject's voxel level tensor estimate to the template where the ROIs are used to generate and calculate the ALPS index in template space.
3. FLIRT → FNIRT → INVWARP. This uses the same registration approach as approach 2 but estimates the inverse warp allowing the template based ROIs to mapped to subject space to calculate the ALPS index.
4. FLIRT → FNIRT → VECREG (instead of APPLYWARP) to register each subject's scan to a template where the ROIs are used to generate and calculate the DTI-ALPS index (i.e. measured in template space). The VECREG warping of the DTI tensor appropriate handles reoriented of the tensor into atlas space where the ALPS index is calculated.

Of the above approaches, 3-4 are the preferred approach and should give highly correlated results. Approach 1 does not fully account for anatomical variability between the subject and atlas while approach 2 does not appropriately handle reorientation of the diffusion tensor.

While clinical studies have eagerly applied the ALPS index to a multitude of distinct conditions, recent methodological and mathematical deconstructions have challenged its biological interpretation. Rather than acting as a direct proxy for dynamic fluid transport or bulk perivascular clearance, the ALPS index has been mathematically reduced to a basic ratio of radial diffusivity asymmetry (the balance between the L2 and L3 eigenvalues of the diffusion tensor).

Because crossing WM fibers occupy up to 90% of WM voxels - and perivascular spaces account for a mere 1% - the metric is heavily dominated by static tissue geometry, fiber dispersion, and local neuroinflammation. This review synthesizes hundreds of ALPS index publications, the vast majority of which were published in 2025. By bridging the microstructural physics of the tensor with macro-level clinical observations, this paper provides a unified framework to explain why DTI-ALPS is ubiquitously altered across disparate neurodegenerative, sleep, and psychiatric disorders, while defining its translational roadmap in future clinical trials.

## 2 Materials and methods

### 2.1 Aims

We aimed to review the literature related to studies using DTI-ALPS to provide a survey of how the technique is being used.

### 2.2 Methods

A review of the literature was performed on 1 October 2025. We searched MEDLINE (OvidSP), EMBASE (OvidSP), BIOSIS Previews (ISI Web of Knowledge), Web of Science and Conference Proceedings (ISI Web of Knowledge), PsycINFO (OvidSP), and LILACS (BIREME), using search terms “dti alp”, “dti alps”, “dti glymphatic”, “dti glymphatics”, “diffusion glymphatic”, “diffusion glymphatics”. No language or date restrictions were applied to the electronic searches and methodological filters were not used so as to maximize sensitivity.

## 3 Results

**Fig. 3:**
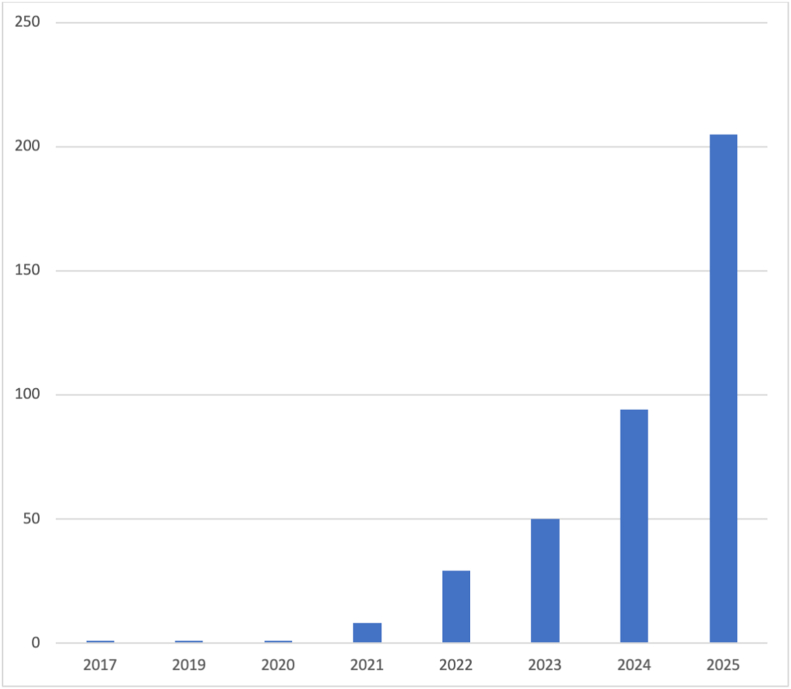
Frequency of ALPS index publications since the ALPS index's inception in 2017.

The search of the literature databases resulted in 393 ALPS index publications. We assessed each and categorized them into Studies or Reviews/Editorials. Each Study was then labelled according to its main research focus. There were one-to-two studies published for each year 2017-2020; as seen in Fig. 3, there has been an exponential growth in the number of annual ALPS index publications since its inception with more than 50% of the findings published in 2025.

**Fig. 4:**
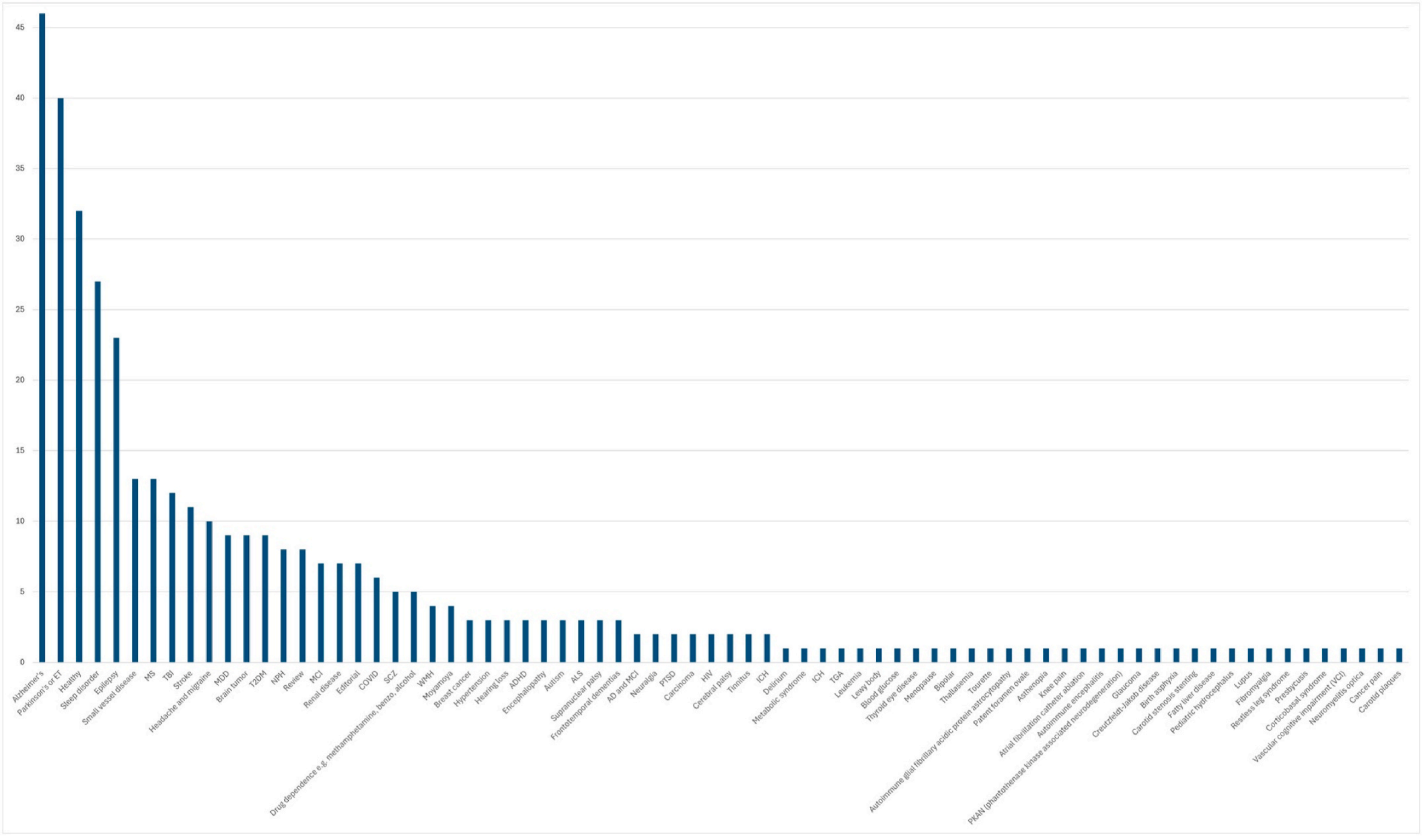
Frequencies of conditions investigated using the ALPS index.

The majority of studies have evaluated the ALPS index across 70 clinical conditions (Fig. 4) while 32 studies reported data only from only a healthy control (HC) population. The 10 most common conditions studied have been in neurological disorders, which have included: Alzheimer's disease (46 publications, as well as nine in mild cognitive impairment); Parkinson's disease/Essential Tremor (40 publications); sleep disorders (27 publications); epilepsy (23 publications); small vessel disease (13 publications); multiple sclerosis (13 publications); traumatic brain injury (12 publications); stroke (11 publications); as well as headache and migraine (10 publications). Seventeen studies have employed the ALPS index to study psychiatric disorders including major depressive disorder, schizophrenia, bipolar disorder, post-traumatic stress disorder, and delirium. Of the 393 publications, fifteen reported longitudinal results. There have been eight reviews and seven editorials published at the time of this query. Twenty-two of the publications have specifically focused on the ALPS index methodology.

## 4 Discussion

There has been an exponential growth in the number of published studies between the initial publication of the method in 2017 and this literature review in 2025. The primary focus of these publications has been using the ALPS index as a putative indicator of glymphatic system health, function, and/or efficiency. The index has been used mostly in disease cohorts of aging-related neurodegeneration (such as Alzheimer's disease, AD) or non-age-related neurodegeneration (such as Multiple Sclerosis, MS). As the GS has a direct putative relationship with sleep, it is unsurprising that many of the ALPS index publications focused on this domain. Studies outside of the neurodegeneration domain have included epilepsy, traumatic brain injury, headache/migraine, as well as various psychiatric disorders including schizophrenia, major depression, and bipolar disorder. The majority of studies reported the ALPS index to be significantly lower in the clinical group as compared to HC groups and associate with disease progression, severity, and age.

In addition to evaluating ALPS index changes with neurodegeneration and disease, several other studies have focused on methodological issues associated with obtaining the ALPS index. They have found that reliable estimation of the ALPS index requires standardized ROI placement and size, MRI acquisition echo time, and head positioning within the scanner. The putative interpretation of the ALPS index as a marker of the GS has also been called into question as the index's calculation may be more representative of underlying WM microstructure than a reflection of the GS. Studies note that the ALPS index does not consider spatial and temporal variations in CSF-ISF exchange throughout the human brain (Ringstad, 2024) since they are based on two small ROIs. It is unlikely that these ROIs represent the GS across the whole brain especially given the fact that perivascular spaces in WM account for only about 1% of the tissue (Barisano et al., 2021). In addition to measuring AQP4 water channel functionality, the ALPS index is purported to also sensitive be to blood–brain barrier integrity and changes in WM microstructure which do not directly reflect GS function (Georgiopoulos et al., 2024; Jeurissen et al., 2013; Schilling et al., 2025; Shirbandi et al., 2025).

We will now discuss the findings of the most studied clinical groups which used the ALPS index, followed by the consideration of white matter hyperintensities. ALPS index in the context of findings in sleep studies will then be reviewed, followed by the results of the index in psychiatric disorders, and the relationship of the index with age. We will then delve further into methodological considerations, including limitations and critiques of the ALPS index technique. We will then briefly review alternate methods for investigating the GS.

### 4.1 Using DTI-ALPS to study neurodegenerative diseases: dementia, Parkinson's disease, multiple sclerosis

It has been reported that the ALPS index is lower in common neurodegenerative diseases such as dementia (the most commonly investigated form was Alzheimer's disease, AD), PD, and MS in cross sectional studies comparing these populations to matched controls (Khalafi et al., 2025; Shirbandi et al., 2025; Tranquille et al., 2026). In addition, the ALPS index has been found to decrease over time in longitudinal studies and hence interpreted as a marker of disease progression (Bayoumi et al., 2024; Jungwon et al., 2025; Wood et al., 2024).

The primary disease studied with the ALPS index has been AD. Khalafi et al. (2025) conducted a meta-analysis of 19 studies which reported the ALPS index in AD, mild cognitive impairment (MCI), and HC groups. They found the overall index was significantly lower in AD subjects as compared to HCs, lower in AD as compared to MCI, as well as lower in MCI versus HC subjects. The ALPS index has been shown to have a significant association with the Mini-Mental Status Examination (MMSE (Folstein et al., 1975);) performance (Burles et al., 2025), and there was a negative correlation between the ALPS index and amyloid deposition on PET (Park et al., 2023). Similarly, Kamagata (Kamagata et al., 2022) reported a lower ALPS index associated with lower CSF Aβ42, lower FDG-PET uptake, as well as worse cognition across several domains. Steward et al. (2021) found significant correlations between ALPS index and ADASCog11 in the right hemisphere after adjusting for age, sex, and APoE ε4 status, in older adults. However, not all studies have observed strong associations with AD progression. In a study consisting of over 2000 subjects, Satpathi (Satpathi et al., 2025) reported low associations with AD biomarkers, suggesting that ALPS index may be a poor marker for AD progression. Furthermore, another study found no significant change in the index after Amyloid-Targeting Disease-Modifying Therapy in a small longitudinal study consisting of 13 patients (Oura et al., 2025). Finally, a post-mortem study by Li et al. (2025) found that the ALPS index was more associated with Aβ pathologic characteristics and age and less with AD markers.

Parkinson's disease has been the second most common illness studied with the ALPS index. In a meta-analysis of 11 studies involving 1462 patients (855 PD, 607 non-PD), it was found that the ALPS index was significantly lower in PD as compared to matched control participants as well as having a significant negative correlation with the Unified PD Rating Scale III (UPDRS III) (Shirbandi et al., 2025). Similarly, in another meta-analysis including 35 studies representing 4224 patients with PD and 1287 healthy controls, Firouzabadi and colleagues found that the ALPS index was significantly correlated with motor symptom severity across studies but not with psychiatric symptoms or gender (Rajai Firouzabadi et al., 2025). In a study employing a 7T MRI scanner, Shen et al. (2022) reported lower ALPS index in 76 PD patients compared to 48 controls, with differences greater in participants with a Hoehn & Yahr stage greater than two (Shen et al., 2022). The lower ALPS index was associated with a longer duration of illness and UPDRS total scores. However, another study found that the ALPS index may not be a sensitive indicator of glymphatic function in the initial stages of neurodegeneration in PD (Georgiopoulos et al., 2024), although this finding may also relate to the methods used to measure ALPS index (see below). In a sample of 165 MS patients it was reported that lower ALPS index values were associated with age, lesion volume and disease duration (Santaniello et al., 2025). Furthermore, in a longitudinal study (Tranquille et al., 2026) it was found that although decreased baseline ALPS index was associated with disability progression in relapsing-remitting MS changes in the ALPS index over five years were not associated with disease progression.

### 4.2 White matter hyperintensities

Ubiquitous to aging is the evolution of white matter hyperintensities (WMHs) which have not always been considered when computing the ALPS index. Furthermore, choroid plexus (CP), which produces most of the CSF and has a larger number of AQP4 channels (MacAulay et al., 2022) is not considered in ALPS index measurement. To this end, Xu and colleagues (Xu et al., 2024) scanned a cohort of 206 participants with known WMHs and 43 controls and found that participants with large WMH volumes and a lower ALPS index had a larger CP volume (CPV) as compared to HCs and those with only mild WMH volumes. They suggest that CPV may be an important variable to consider when studying the GS as it has been positively associated with severity of cognitive impairment in neuropsychiatric disorders (Althubaity et al., 2022; Lizano et al., 2019) and neurodegenerative diseases (Choi et al., 2022; Jeong et al., 2023). Enlargement of the CP has been linked with inflammation (Althubaity et al., 2022; Lizano et al., 2019) and glymphatic dysfunction (Jeong et al., 2023; Li et al., 2023), which may lead to WMHs and cognitive impairment (Hannawi, 2024).

Utilizing data from three independent studies, Schirge et al. (2025) reported that AD patients had a significantly lower ALPS index then healthy controls, the ALPS index was associated with CSF Aβ concentration, WMH number, MMSE and Clinical Dementia Rating (CDR (Morris, 1993);) scale (Schirge et al., 2025). In another study, Jeong (Jeong and Choi, 2024) found a significant negative correlation between WMH and ALPS index, but only in those aged 50-59. The ALPS index has also been shown to negatively correlate with WMH volumes (Chen et al., 2025a) and baseline ALPS index to be negatively associated with WMH progression at follow-up.

### 4.3 Sleep

It has been shown that there is a 60% increase in interstitial volume during sleep, which results in a large increase in the CSF-ISF exchange and extracellular solute removal (Xie et al., 2013); the ALPS index, as a putative measure of the GS, would therefore be expected to be associated with sleep patterns or disturbances. A recent systematic review of severe obstructive sleep apnea (OSA) identified four studies (137 patients with severe OSA and 170 HCs) (Ghaderi et al., 2025) and found a significantly lower ALPS index in severe OSA patients. Similarly, Saito et al. (Saito et al., 2023a,b,c) reported that the ALPS index was significantly lower in sleep disruption. Furthermore, they found that the ALPS index was significantly negatively correlated with the PSQI (Pittsburgh Sleep Quality Index) total scores as well as the sub-components of sleep latency and use of sleep medication.

An important validation of using the ALPS index as biomarker for glymphatic function is the observation of changes in ALPS index during sleep. To this end, Yun et al. (2025) studied nine subjects once while awake and 16 times during sleep. They found that the ALPS index significantly increased within 30 min of sleep induction supporting the hypothesis that it is a potential biomarker for glymphatic function. However, this study did not find the expected increase in the x-direction diffusivity during sleep that was thought to reflect changes in glymphatic activity. This suggests that if the ALPS index reflects glymphatic activity other measures obtained from the diffusion data are likely associated with such activity. Another study by Ko and colleagues (Ko et al., 2025) scanned 539 healthy individuals during various times of the day and night. They found that the ALPS index varies across the day. These findings are consistent with Saito et al. (Saito et al., 2023a,b,c) who observed lower ALPS index in those with sleep disruption as well as Lee et al. (2021) who demonstrated increased gadolinium clearance after sleep. These findings emphasize the importance of circadian timing in evaluating glymphatic function using the ALPS index. In addition, a previous study has shown that genetic deletion of AQP4 disrupts the circadian regulation of CSF distribution (Hablitz et al., 2020). Moreover, the suprachiasmatic nucleus, the brain's central clock, plays a key role in regulating astrocytic gene expression patterns and maintaining rhythmic glymphatic function (Tso et al., 2017). This suggests that astrocytes synchronize glymphatic activity with the circadian cycle.

In contrast to studies described above, Han et al. (2023 (Han et al., 2023);) reported no circadian rhythm dependence of the ALPS index in the awake state from 8:00 to 23:00, although their sample size was small (N = 22).

### 4.4 Psychiatric disorders

There have been at least 17 published ALPS index studies of individuals with mood disorders, including major depressive disorder (MDD), schizophrenia (SCZ), bipolar disorder, and delirium. Most reported consistent findings of lower ALPS index in the clinical groups when compared with HCs.

In MDD, it has been reported that the ALPS index is lower than matched controls at baseline and increased with treatment of vortioxetine Guo et al. (2025). Yang et al. found the ALPS index to be negatively correlated with depression severity (Yang et al., 2025), which is consistent with other ALPS index studies of MDD such as those by Chen et al. (Chen et al., 2025b), Yang et al. (2024), and the large study by Gong et al. (2025) which included 665 MDD patients and 338 HCs. In contrast, Zhang et al. (2025) reported no significant differences in the ALPS index in a study of major depression. Their results suggest that the GS may not contribute to its pathogenesis in the same way as in adults as the cohort was of younger (adolescent) subjects. However, this was one of the only studies to have manually drawn the ROIs onto each scan as opposed to using previously published coordinates to automatically create the ROIs. Hence, it is possible that this difference in methodology may partially explain their negative findings.

In a study of 43 people with SCZ and 108 HCs, Tu and colleagues (Tu et al., 2024a,b) reported significantly reduced ALPS index which also correlated with cognitive performance, consistent with Wu et al. (2025). Abdolizadeh et al. (2024) recruited 103 patients with SCZ and 47 matched HCs and found that the ALPS index was lower in patients compared with HCs, and illness duration was negatively correlated with the right ALPS index.

Kikuta and colleagues found a reduced ALPS index in patients with bipolar disorder when compared with HCs (Kikuta et al., 2025), consistent with Ueda et al. (2024). Tu et al. (Tu et al., 2024a,b) reported that patients with delirium exhibited significantly lower ALPS indexes compared to HCs.

### 4.5 Relationship with age

Another consistent finding in the literature is that ALPS index is significant correlated with age. In a large study from the UK Biobank participants (n = 17723), Clark et al. found the predicted associations between the ALPS index and age, sleep, cognitive performance, and sex suggesting that the ALPS index is a stable measure consistently associated with demographics, sleep, and cognition (Clark et al., 2024). Yin et al. studied 205 subjects aged 40-82 divided into subgroups by age and found that ALPS index negatively correlated with age only in the group who were over 65 years (Yin et al., 2025). In a smaller study (N = 49), Ozsahin et al. also found that a lower ALPS index was associated with age (Ozsahin et al., 2024). They also found that there was a significant difference in the ALPS index between men and women with men having a lower ALPS index. They also found a significant negative correlation between ALPS index and intracranial volume (ICV), suggesting that the differences in ICV could be a confounding factor.

### 4.6 Differences in methodology

The variation in the definition (size, shape, placement) of the ROIs used to measure the ALPS index across studies has been aptly described by Ulloa et al. (2025). For example, Taoka et al. compared the ALPS index estimated from various ROI configurations by generating the index from a variety of ROIs consisting of various sizes placed parallel and orthogonal to the lateral ventricle (Taoka et al., 2022). They found significant differences in the generated ALPS indexes with different ROI configurations, demonstrating the importance of standardizing the ROI characteristics for consistent ALPS index calculation and is consistent with findings and suggestions from other ALPS index studies (Qiu et al., 2025; Taoka et al., 2024). While 22 papers have focused on ALPS index methodology, many of the other articles discussed the technique and some of the variations that may contribute towards the estimation and interpretation of the index.

From the lab which created the ALPS index (Taoka et al., 2017), Taoka and colleagues published a comprehensive overview of the use of ALPS index covering literature published until 2024 which related the index to various conditions including AD, PD, small vessel disease, iNPH, TBI, demyelinating disease, sleep, and aging (Taoka et al., 2024). Due to at least 50% of ALPS index studies being published in 2025, their overview was unable to include a large number of findings reported in the last year which account for almost 50% of the published literature on this topic. However, they acknowledged that there are problems pertaining to the interpretation of the ALPS index and stated that “a higher ALPS-index indicates predominant Brownian motion of water molecules in the radial direction at the lateral ventricular body level, no more and no less”. They suggested that the ALPS index should simply be expressed as high or low, but its interpretation in the context of the GS “is better to be discussed carefully”, and that it may not represent the entirety of the GS. They also asked users to pay particular attention when placing the ROIs to avoid mixing of subcortical fibers whose main axis is in the x-direction, otherwise the ALPS index will be inflated.

Hence, standardization is the key to consistency. Qiu et al. assessed interrater reliability among neuroradiologists in native space compared to ALPS indexes derived from ROIs placed in scans collected from 16 healthy control participants versus standardized ROIs in MNI space (Qiu et al., 2025). They reported only moderate to good intraclass correlation coefficients as well as significant differences between native and MNI space measurements for left-sided ALPS indices, demonstrating the importance of standardized ROI selection approaches for clinical applications of DTI-ALPS.

There are several factors related to calculation of the ALPS index in addition to the ROI size and placement, such as head position, TE (echo time), and number of diffusion directions. For example, Taoka et al. pointed out the axial slice must be perpendicular to the projection fiber at the time of imaging - head position (and therefore imaging plane) has an impact on the ALPS index. These findings were consistent with their earlier study (Taoka et al., 2022) which examined an automated ALPS index pipeline in a study called CHanges in Alps index on Multiple conditiON acquIsition eXperiment (CHAMONIX) which aimed to evaluate the reproducibility of the ALPS index method and changes in the index due to different imaging conditions or calculation methods. They found the ALPS index to be robust under the fixed imaging method even when different scanners were used. However, the index was influenced by the imaging plane, the number of diffusion gradient directions, and TE used. Similarly, Tatekawa et al. also reported that head position correction led to a more reproducible ALPS index using data from 234 cognitively normal subjects from the OASIS-3 dataset (Tatekawa et al., 2023). In addition, studies evaluating registration techniques for obtaining the ALPS index have observed that variations in head orientation can substantially affect the validity of ALPS index calculations (Ajouz et al., 2026; Burles et al., 2025), which is consistent with the study by Taoka (Taoka et al., 2022).

Liu et al. (2024) acquired DTI data on 50 subjects scanned on 3T MRIs from three major scanner vendors (GE, Siemens, Philips) at seven sites and reported high ALPS index inter-scanner reproducibility (ICC = 0.77 to 0.95, p < 0.001), inter-rater reliability (ICC = 0.96 to 1, p < 0.001) and test-retest repeatability (ICC = 0.89 to 0.95, p < 0.001). Furthermore, Saito et al. (Saito et al., 2023a,b,c) processed DTI data of 45 patients with AD and 82 cognitively normal (CN) participants from the ADNI database, acquired from 27 institutions and three different 3T MRI scanners. They employed the COMBined Association Test (COMBAT) technique that was able to harmonize ALPS index variations caused by scanner, site, and protocol differences thus facilitating the use of the ALPS index in multi-center studies.

In addition to subject positioning and TE used for the imaging protocol, it is possible that different b-values (strength of diffusion sensitization) may have an impact upon the inferred interpretation of the ALPS index as an indicator of GS integrity. Most studies used a b-value of b = 1000 s/mm^2^ but it unknown whether this is the optimal value to investigate the GS. Furthermore, Schilling et al. (2025) demonstrated that the ALPS index changes with different b-values. In addition, Demiral et al. demonstrated that slow and fast apparent diffusion coefficients derived from low b-values provide a more accurate reflection of GS functionality, although b-values of 2000 s/mm^2^ or higher might allow for greater sensitivity to tracking ISF changes (Demiral et al., 2019).

Han et al. (2023) measured GS influx using ultra-long TE-low-b-value (b = 130 s/mm^2^) based on the method introduced by Harrison et al. (2018), and GS efflux using ALPS index at a conventional b-value of 1000 s/mm^2^. They reported that the GS influx rate was higher and the efflux rate was lower in those older than 45 years. They suggested that GS differences might be due to age-related changes in arterial pulsation and AQP4 polarization.

The presence of lesions is another factor that would be expected to affect the ALPS index, and accurate placement of the ROIs to reliably capture PVSs would improve its reliability and interpretability as demonstrated by Carotenuto (Carotenuto et al., 2022). Additionally, some studies utilized susceptibility weighted imaging (SWI) scans (e.g. (Cai et al., 2025; Ulloa et al., 2025)) to identify the location of the medullary veins, which may enhance the accuracy of ALPS index ROI placement (Yun et al., 2025). However, Taoka et al. (2022) reported high test-retest reproducibility of the ALPS index without SWI for ROI placement. Rather than relying on atlas based ROI definitions to accurately capture the region of project and association fibers, automated techniques to locate PVSs may be preferable. For example, Gunter and colleagues demonstrated fully automated probabilistic PVS segmentation from basic clinical MRI sequences including T1-weighted, T2-weighted, and FLAIR images (Gunter et al., 2023).

Other factors that may influence the ALPS index are the resampling approaches as well as the inclusion/exclusion of voxels with zero values. Saito et al. scanned 23 participants twice (each on the same day) and reported automated the ALPS index had higher reproducibility when using VECREG for resampling the tensor field as compared to APPLYWARP (Saito et al., 2023a,b,c) since it is properly rotating the diffusion tensor. Due to errors in the tensor fitting it is possible to generate voxels with zero values. Not ignoring zero values would easily deflate ROI metrics but whether zero values are more likely to be appear in the numerator (Dxx association and Dxx projection) or denominator (Dyy projection and Dxx association) ROIs is unknown, hence further highlighting the importance of excluding (ignoring) zero values when calculating the ALPS index. In summary, variations in scanner vendors, head positions, diffusion protocols, ROI definitions, and analysis approaches across studies all have an impact on the resulting ALPS index measured and contribute to masking of true disease-related declines.

### 4.7 Limitations and critiques of DTI-ALPS

Can a technique such as diffusion tensor imaging designed to investigate WM microstructure be used as a measure of the GS? Furthermore, the extent to which glymphatic clearance contributes to total brain clearance capacity in general remains uncertain, since spatial and temporal variations in CSF-ISF exchange throughout the human brain have been demonstrated (Ringstad, 2024). Hence, careful interpretation is required to determine its relevance to the overall GS function (Abdolizadeh et al., 2024). After all, it is simply based on two relatively small ROIs at one axial slice where diffusivity only along medullary veins is assessed and does not measure the GS across the whole brain (Ozsahin et al., 2024). Therefore, the big question that needs to addressed is how well does changes in fluid motion in this small region reflect the overall the clearance capacity of the brain (Agarwal, 2025).

As PVS in WM account for only about 1% of the tissue it is therefore likely that the ALPS index is not measuring only PVS around the deep medullary vein but a large portion of the measure reflects the surrounding WM tissue microstructure (Andica et al., 2023; Barisano et al., 2021). Furthermore, patient motion and blood flow are also confounding factors (Ringstad, 2024), as well as AQP4 water channel functionality and blood–brain barrier integrity (Shirbandi et al., 2025).

As pointed out by Satpathi (Satpathi et al., 2025), only a small fraction of the total waste clearance occurs in the deep WM (which is where the ALPS index is measured) whereas glymphatic activity is primarily observed near the brain's surface (Ringstad, 2024), and little signal would be detected given the size of MRI voxels (in mm) when compared to the region being studied (which is in the order of microns). This is especially true when considering the low signal that would be detected at b = 1000 in the context of the isotropic diffusion speed. Satpathi and colleagues also found the ALPS index had a poor association with amyloid deposition as well as overall and regional PVS load (Satpathi et al., 2025).

The GS is a complex system that includes several types of glymphatic flow (cardiac impulse, respiratory pulse, low-frequency wave, and shallow-wave frequency propagations) and several vital processes (including paravascular transport, interstitial fluid transport, bulk cerebral CSF flow, meningeal lymphatic efflux, dynamic cerebral mantle pulsations, bulk spinal CSF flow, and spinal CSF efflux) potentially requiring multiple approaches to fully understand its function (Jessen et al., 2015; Klostranec et al., 2021).

### 4.8 Crossing white matter fibers

Georgiopoulos et al. demonstrated that the ROIs used to measure the ALPS index containing crossing WM fibers significantly inflate the index (Georgiopoulos et al., 2024). Similarly, Schilling et al. (2025) systematically investigated the impact of crossing fibers upon the ALPS index. They found a dominant contribution from axonal geometry rather than faster PVS-specific diffusion and that crossing fibers significantly inflate ALPS indices, with greater radial asymmetry observed in regions with a greater prevalence of crossing fibers. This is a key point as crossing fibers are present in up to 90% of WM voxels (Jeurissen et al., 2013; Schilling et al., 2022) and, as mentioned above, PVS in WM account for only about 1% of the tissue (Barisano et al., 2021). They also reported that anisotropic axonal dispersion and undulations (a waviness pattern of axonal trajectories observed in histological preparations) introduce systematic asymmetry independent of perivascular diffusion. They also found that high-resolution vascular imaging reveals substantial heterogeneity in medullary vein orientation, challenging the assumption that PVS consistently aligns with the left-right axis in ALPS regions. Hence, ALPS index metrics may not provide a direct measure of glymphatic function but rather reflect underlying axonal geometry. Because the ALPS index is influenced by underlying axonal geometry and radial diffusivity asymmetry, any disease that degrades WM microstructure or causes neuroinflammation will alter the ALPS index. This directly explains why alterations are observed across 70+ seemingly unrelated conditions (e.g. neurodegenerative, psychiatric, and sleep disorders).

### 4.9 Is DTI-ALPS simply measuring radial diffusivity asymmetry?

Mathematically, the calculation of the ALPS index (equation (1)) can be reduced to simply represent the ratio of second (L2) and third (L3) eigenvalues of the diffusion tensor. Therefore, equation (1) can be rewritten as

(2) ALPS index = (L2 + L2) / (L3 + L3) => L2 / L3

Hence, the ALPS index is a measure of the degree of radial diffusivity asymmetry as described by a number of studies (Georgiopoulos et al., 2024; Satpathi et al., 2025; Schilling et al., 2025; Wright et al., 2024)). The ALPS index significantly correlates with radial diffusivity asymmetry and age significantly correlates with L2/L3 (Schilling et al., 2025) (F ig. 7). The observed ALPS index can therefore be attributed to the radial asymmetry of projection and association tracts, rather than solely originating from the diffusivity within the PVS (Wright et al., 2024). While the ALPS index depends on the assessment of white matter architecture, it may be the case that this underlying microstructure impacts the perivascular space, extracellular space, and ultimately fluid mobility. While it is unlikely that DTI-ALPS is a direct measurement of glymphatic clearance it likely provides biologically meaningful information about the local tissue milieu and its relation to interstitial fluid dynamics.

### 4.10 Alternative methods of investigating the glymphatics system

Due to the simplicity of ALPS index it has been rapidly adapted in various conditions. There is a variety of direct clinical techniques by which to depict different aspects of the complex GS (Boyd et al., 2024), including via visualization of CSF transport using postcontrast imaging techniques following intravenous or intrathecal administration of contrast material and indirect glymphatic assessment with detection of enlarged PVSs (Klostranec et al., 2021). While these direct techniques represent the most accurate method for assessing glymphatic function, the inherent invasiveness of these approaches has spurred increasing interest in the development of non-invasive MR-based alternatives (You et al., 2025) such as ALPS index, which has been shown to have a strong correlation with intrathecal contrast administration for the evaluation of GS function (Zhang et al., 2021). Other non-invasive MRI-based methods include intravoxel incoherent motion (Wong et al., 2020), CSF flow and CSF production (Pomerantz, 2016), AQP4 channel activity (Ohene et al., 2019), meningeal lymphatic vessels (Zhou et al., 2020), CEST (chemical exchange saturation transfer) (Zhang et al., 2023), PI (pulsatility index) of cortical arteries at the centrum semiovale level (PICSO)(You et al., 2025), and tissue-to-fluid water-exchange imaging using T2-selective saturation labeling (Taso and Alsop, 2025). Direct methods (DCE-MRI) are preferred for active macrovascular fluid clearance tracking, whereas the ALPS index is preferred for non-invasive, large-scale psychiatric or longitudinal monitoring. They are complementary but decoupled (Mossige et al., 2026), as one measures active fluid transport and the other measures static tissue environment boundaries. Table 1 categorizes the distinct preferences of alternative glymphatic metrics, evaluates their comparability, and provides a clear interpretive framework for when these measures contradict one another within the same disease state.

**Table 1:** A comparative table of glymphatic assessment method preferences, comparability, and inconsistency interpretation.

Glymphatic Assessment Method	Primary Physiological Target	Clinical Context Preferences	Comparability to DTI-ALPS	Interpretation of Inconsistency (e.g., Target Method is Abnormal, but DTI-ALPS is Normal)
**DTI-ALPS Index**	Radial diffusivity asymmetry; static white matter geometry; local fluid mobility	Large-scale biobanks, psychiatric cohorts, pediatric/adolescent studies, and retrospective trials where contrast is restricted	**Indirect Reference Component**. Tracks the architectural terrain surrounding fluid channels rather than active bulk flow	Indicates a functional deficit in active fluid clearance or AQP4 channel polarization *before* structural axonal degradation or neuroinflammation has occurred
**Post-Contrast Imaging (Intrathecal/IV DCE-MRI)**	Dynamic tracer transport; macro-fluid influx and bulk parenchymal clearance speed	Severe neurodegenerative disorders, Normal Pressure Hydrocephalus, and acute surgical path-finding where macro-clearance is critical	**Complementary but Decoupled**. Captures macrovascular dynamic clearance velocity, whereas ALPS measures local microscopic water diffusion	Indicates that white matter structural geometry remains intact, but global CSF-ISF exchange/bulk driving pressures (e.g., arterial pulsation) are actively failing
**Phase-Contrast MRI/Cortical Pulsatility (PICSO)**	Bulk CSF flow velocity; macromolecular arterial drive and pulsatility indices	Cerebrovascular small vessel disease, vascular dementias, and sleep state transition tracking	**Mechanistic Component**. Measures the physical "pump" that forces glymphatic flow, while ALPS measures the structural response of the tissue	Indicates a physiological mismatch where the physical driving force (pulse wave) is altered, but the local deep white matter tissue environment has not yet remodeled
**Ultra-Long TE, Low b-value Diffusion MRI**	Perivascular space fluid influx rate separate from efflux	Age-related degeneration mapping and longitudinal tracking of early vascular aging	**Direct Physiological Axis**. Isolates directional fluid shifts rather than being heavily confounded by crossing axonal fibers	Demonstrates an asymmetry in glymphatic dynamics where fluid influx pathways are hyper-permeable or disrupted, but deep white matter venous efflux structural alignment is stable

### 4.11 ALPS index in translational research and clinical trials

The ubiquity of ALPS index alterations across more than 70 clinical conditions highlights its role as a highly sensitive, albeit non-specific, marker of WM microstructural alteration, local neuroinflammation, and fluid dynamic disruption. The ALPS index captures shared macro-structural endpoints (axon bundle degeneration, extracellular edema, and neuroinflammation) rather than a disease-specific pathway. Rather than limiting its utility, this broad sensitivity provides distinct advantages for translational research and clinical trial design. Because the ALPS index requires only standard single-shell diffusion data with relatively low b-values (b = 1000 s/mm^2^), it can be calculated retrospectively from vast, existing neuroimaging biobanks. In translational workflows, this allows researchers to instantly screen and stratify large patient cohorts at an epidemiological level (Lin et al., 2026) at no additional imaging cost. For instance, individuals exhibiting exceptionally low baseline ALPS indices within an aging or prodromal population can be selectively fast-tracked into expensive, targeted clinical trials involving invasive or high-cost specific biomarkers (such as tau/amyloid PET or CSF assays) and has recently been suggested as potentially a clinical risk-stratification tool (Dehaghi et al., 2026), regardless of whether its origin is purely fluid or microstructural (Grigoras et al., 2025). However the ALPS index is not a standalone diagnostic tool, but a rapid, low-cost, non-invasive retrospective screening tool that should be combined with higher-specificity markers (like plasma biomarkers or PET) for clinical trial stratification.

The non-invasive and highly reproducible nature of automated ALPS index processing makes it an ideal surrogate endpoint for tracking longitudinal treatment efficacy. As clinical trials shift toward preventive interventions - such as evaluating the impact of novel sleep apnea therapies or psychiatric treatments like vortioxetine - the ALPS index provides a safe, repeatable window into whether normalizing a patient's clinical state successfully translates to a restoration of the local tissue milieu and fluid mobility. Already there are efforts to tweak the traditional ALPS index formula to improve clinical interpretation (Jamali et al., 2026).

## 5 Conclusion

DTI-ALPS is a simple, efficient, safe and non-invasive technique which generates an index shown to significantly associate with aging and differentiate an array of clinical conditions from healthy subjects. Although there is some evidence that the index correlates with conventional invasive measures of the glymphatics system, methodological investigations have demonstrated that the index is likely a composite representation of white matter structure, perivascular diffusion, extracellular space, and local fluid mobility. Furthermore, a variety of acquisition and analysis parameters impact the ALPS index. As such, it is imperative that data acquisition and analysis methodology is standardized in order to obtain consistent DTI-ALPS metrics. While direct methods (DCE-MRI) are preferred for active macrovascular fluid clearance tracking, DTI-ALPS is suitable for non-invasive, large-scale psychiatric or longitudinal monitoring.

## Declarations

The authors have no relevant financial or non-financial interests to disclose not any competing interests to declare.

## Ethics statements

This study does not include new human-subject experiments. All procedures were performed in compliance with relevant laws and institutional guidelines and have been approved by the appropriate institutional review board (IRB).

## CRediT authorship contribution statement

**Jerome J. Maller:** Conceptualization, Data curation, Formal analysis, Investigation, Writing – original draft, Writing – review & editing. **Vincent A. Magnotta:** Conceptualization, Methodology, Writing – original draft, Writing – review & editing.

## Declaration of competing interest

The authors declare the following financial interests/personal relationships which may be considered as potential competing interests: The authors have no financial interests/personal relationships which may be considered as potential competing interests.

## Data Availability

Data will be made available on request.
